# Extended State Observer-Based Chattering Free Terminal Sliding-Mode Control of Hydraulic Manipulators

**DOI:** 10.3390/s25216787

**Published:** 2025-11-06

**Authors:** Han Gao, Jingran Ma, Yanjun Liu, Gang Xue

**Affiliations:** 1 Key Laboratory of High Efficiency and Clean Mechanical Manufacture of Ministry of Education, National Demonstration Center for Experimental Mechanical Engineering Education, School of Mechanical Engineering, Shandong University, Jinan 250061, China; 202320665@mail.sdu.edu.cn; 2 Institute of Marine Science and Technology, Shandong University, Qingdao 266237, China; 202236980@mail.sdu.edu.cn

**Keywords:** hydraulic manipulator, electro-hydraulic servo system (EHSS), extended state observer (ESO), terminal sliding-mode (TSM)

## Abstract

High-performance tracking control for the hydraulic manipulator should address the challenges of the uncertainties and unknowns associated with the electro-hydraulic servo system (EHSS). This paper presents an extended state observer-based chattering-free terminal sliding-mode (ESO-CFTSM) control scheme for hydraulic manipulators. A third-order integral chain model is developed to characterize the system dynamics, where uncertainties and unknowns are considered as disturbances and estimated by the ESO. Meanwhile, a full-order TSM manifold is designed to stabilize the closed-loop system in finite-time. For this proposed scheme, the feedforward compensation of disturbances is introduced in the equivalent control law. Furthermore, the composite reaching law and a low-pass filter are used to realize the chattering-free control. The singularity is avoided because there are no derivatives of terms with fractional powers in the control law. The stability of the overall system is proved by Lyapunov technique. The simulations using the physical model of a hydraulic manipulator with coupled dynamics show the effectiveness of the proposed scheme for trajectory tracking problems. Simulation results indicate that the proposed ESO-CFTSM can achieve superior performance without being affected by lumped disturbances.

## 1. Introduction

Hydraulic manipulators are usually driven by the electro-hydraulic servo system (EHSS), and they have become the main operating mechanism for unmanned, intelligent, and heavy-duty operations [1,2,3]. However, the practical implementation of high-performance tracking control for hydraulic manipulators is constrained by a series of issues, including the uncertainties, nonlinearities and coupling effects [4,5]. Much research has been conducted to address this challenge with different control schemes proposed, such as active disturbance rejection control [6,7], neural network control [8,9], and sliding-mode control [10,11]. Most of the existing works focus on the improvement of modeling accuracy in hydraulic manipulator dynamics, while the entire dynamics of hydraulic manipulators are very complicated and difficult to be modeled due to uncertainties and unknowns. Therefore, the development of high-performance controllers based on system dynamics with uncertainties and unknowns deserves further exploration.

Sliding-mode control (SMC) can provide rapid convergence and high robustness to disturbances. Furthermore, it can be designed without the precise plant model. Depending on the type of sliding surface, SMC can be divided into linear sliding-mode (LSM) control and terminal sliding-mode (TSM) control [12,13]. Due to the benefits of finite-time convergence, TSM control has attracted wide attention [14]. However, the singularity and chattering problems can hinder the industrial applications of TSM control. To avoid the singularity, a simple nonsingular TSM manifold was proposed by adjusting the combination of system states and the values of the power exponents [15], but it is only applicable to second-order systems. On the study of nonsingular TSM manifolds for higher-order systems, inspired by the design of finite-time-stable systems in [16,17], a solution based on integral sliding mode was provided in [18], and a full-order nonsingular TSM manifold was constructed in [19]. Another reaching law for the full-order nonsingular TSM was developed and applied to load frequency control in [20].

Chattering is more difficult to avoid than singularity in SMC, and it has a direct effect on the service life of the components and equipment. Several methods of chattering reduction have been proposed, such as boundary layer methods [21], filtering methods [22,23], and disturbance estimation methods [24,25,26]. Boundary layer methods are performed by placing a small boundary layer with a continuous function near the sliding surface, but they only guarantee that the system states converge into the boundary layer, which will increase the steady-state errors. Filtering methods can smooth the control signal by using a low-pass filter, but the difficulty is to prove the stability of the SMC system with filters. Disturbance estimation methods can reduce the switching gain in SMC by employing a disturbance observer for disturbance compensation.

SMC is essentially a state feedback control method, and the performance improvement of state feedback controllers for the EHSS faces issues such as the accurate estimation of unmeasurable states and compensation of disturbances. The extended state observer (ESO) has the ability to estimate states and disturbances for integral chain systems, while requiring little model information [27]. ESO-based controllers have been widely studied and applied due to the above advantages [28,29]. An ESO-based backstepping controller, where the ESO bandwidth was determined by combining dynamic performance with the maximum load capacity, was applied to a 2-degrees of freedom (DOF) hydraulic manipulator [30]. A reduced-order model of the EHSS was obtained, and the active disturbance rejection control method was proposed based on this model [31]. For hydraulic linear actuators subjected to large external force disturbances, a large switching gain needs to be set in conventional SMC, which can easily lead to chattering. To solve this issue, ESO-based SMC is an effective solution [32,33]. An ESO-based SMC method was developed for the EHSS of marine stabilized platforms, and the desired performance was achieved under disturbances and perturbations [34]. Another ESO-based SMC was developed for the EHSS of legged robots, and a method for tuning parameters was given in [35].

Inspired by earlier works, a novel ESO-based chattering-free TSM (ESO-CFTSM) controller is proposed for the EHSS of the hydraulic manipulator in this paper. The main contributions of this paper are as follows. The composite control scheme of the ESO and chattering-free TSM is found to be applicable to the EHSS, which has the integral chain structure with matched disturbances. Meanwhile, thanks to the nonsingular and chattering-free control, the controller parameters can be easily tuned without making the hydraulic components fail. Moreover, excellent tracking performance can be achieved by the compound motion of hydraulic cylinders.

To this end, the third-order integral chain model for the EHSS of the hydraulic manipulator is first constructed. Secondly, the unmeasurable states and disturbances of the EHSS are estimated by the ESO. Thirdly, a novel chattering-free TSM controller with disturbance compensation is constructed. Finally, simulation results verify the effectiveness and superiority of the proposed controller.

The remainder of this paper is organized as follows. The plant description and model of the EHSS are given in Section 2. The ESO and the ESO-CFTSM controller are developed in Section 3, and the stability analysis is given. The simulation results are presented in Section 4. Finally, the conclusion is drawn in Section 5.

## 2. Plant Description and Model of the EHSS

**Figure 1:**
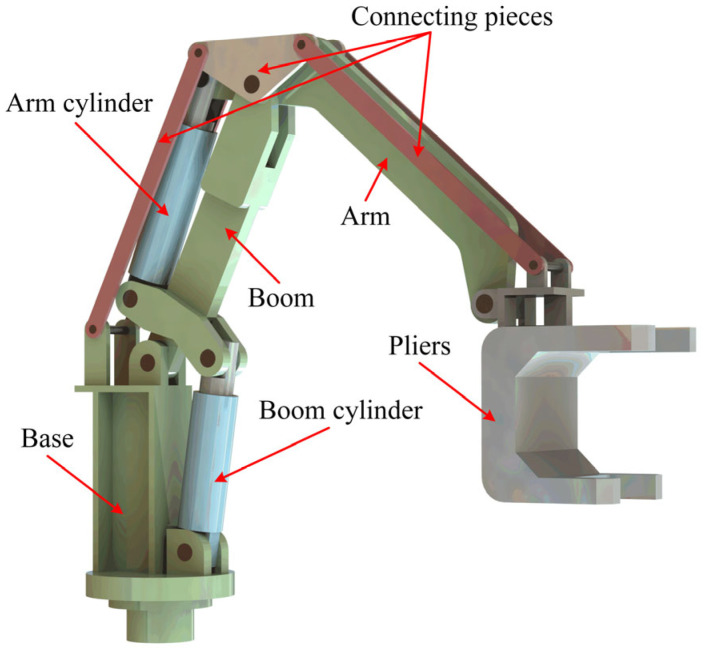
Structure of the HIR.

The mechatronics plant is a 2-DOF hydraulic manipulator called the hydraulic iron roughneck (HIR), as shown in Figure 1. The HIR has a series manipulator that includes a boom and an arm driven by hydraulics and pliers, as well as the connecting pieces that link them together. The pliers are the actuator with large inertia characteristics, whose position in the workspace is determined by hydraulic cylinders, and their work task is to complete the make-up and break-out of drill strings during the hoisting or running operation of the oil drilling and workover.

**Figure 2:**
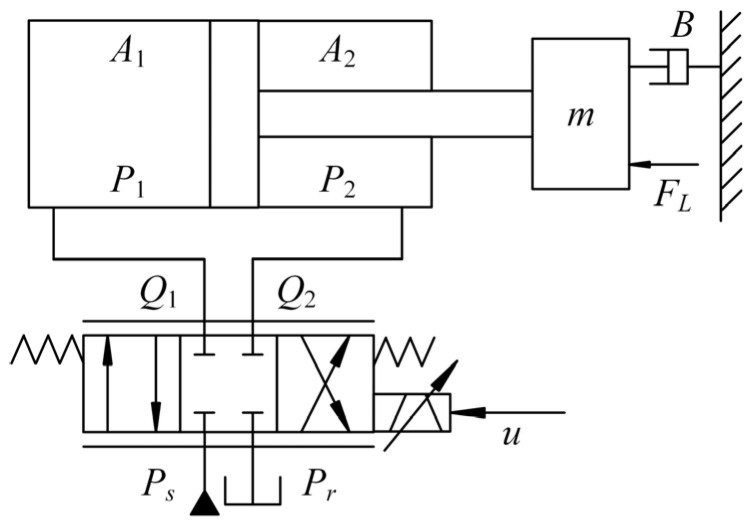
Architecture of the EHSS.

The HIR is driven by an EHSS. In the EHSS, a fixed-displacement pump driven by an asynchronous motor is used as the energy source, a relief valve is used to regulate the supply pressure, and two servo valves are used to distribute hydraulic fluids and realize the position control of the cylinders. The schematic structure of the EHSS is shown in Figure 2.

The load dynamics can be expressed as

(1) $$A_{1}P_{1}-A_{2}P_{2}=m{\ddot{x}}_{p}+B{\dot{x}}_{p}+F_{L}\left(t,x_{p},{\dot{x}}_{p}\right)$$

where $x_{p}$, ${\dot{x}}_{p}$, and ${\ddot{x}}_{p}$ are the position, velocity, and acceleration of the load, respectively; *P*_1_ and *P*_2_ are the pressures inside two chambers of the cylinder; *A*_1_ and *A*_2_ are the effective working areas of two chambers; *m* is the mass of the inertial load, and *B* is the viscous damping coefficient; $F_{L}\left(t,x_{p},{\dot{x}}_{p}\right)$ represents the modeling error as well as uncertain and unknown force disturbances, which contain the coupled dynamics between moving components of the HIR.

Neglecting the external leakage, the flow continuity equation of the cylinder can be written as

(2) $$\left\{\begin{array}{c} {\dot{P}}_{1}=\frac{\beta_{e}}{V_{1}}\left[-A_{1}{\dot{x}}_{p}-C_{i}\left(P_{1}-P_{2}\right)+Q_{1}\right]\\ {\dot{P}}_{2}=\frac{\beta_{e}}{V_{2}}\left[A_{2}{\dot{x}}_{p}+C_{i}\left(P_{1}-P_{2}\right)-Q_{2}\right] \end{array}\right.$$

where $V_{1}=V_{01}+A_{1}x_{p}$ and $V_{2}=V_{02}-A_{2}x_{p}$ are the total hydraulic compressible volumes of the piston and rod chambers; *V*_01_ and *V*_02_ are the initial hydraulic compressible volumes of two chambers; *β_e_* represents the effective bulk modulus; *C_i_* is the internal leakage coefficient of the cylinder; *Q*_1_ and *Q*_2_ are the flows of two chambers.

Define the function $s\left(*\right)$ as $s\left(*\right)=\left\{\begin{array}{c} 1,\;*\geq 0\\ 0,\;*<0 \end{array}\right.$, then the flow equation of servo valves is given as

(3) $$\left\{\begin{array}{c} Q_{1}=\alpha x_{\nu }[s\left(x_{\nu }\right)\sqrt{P_{s}-P_{1}}+s\left(-x_{\nu }\right)\sqrt{P_{1}-P_{r}}]\\ Q_{2}=\alpha x_{\nu }[s\left(x_{\nu }\right)\sqrt{P_{2}-P_{r}}+s\left(-x_{\nu }\right)\sqrt{P_{s}-P_{2}}] \end{array}\right.$$

where $\alpha =C_{d}w\sqrt{2/\rho }$; *C_d_* is the flow coefficient; *w* is the area gradient of valves; *ρ* is the oil density; *x_v_* is the spool displacement; *P_s_* and *P_r_* are the supply and return pressures, respectively.

Considering the response of servo valves used in the study is much higher than the system dynamics, the linear dynamics of valves is described by

(4) $$x_{\nu }=k_{\nu }u$$

where *k_v_* is the servo valve gain, and *u* is the control output.

For simplicity, define $\gamma =\alpha k_{\nu }$ and $\left\{\begin{array}{c} R_{1}=s\left(u\right)\sqrt{P_{s}-P_{1}}+s\left(-u\right)\sqrt{P_{1}-P_{r}}\\ R_{2}=s\left(u\right)\sqrt{P_{2}-P_{r}}+s\left(-u\right)\sqrt{P_{s}-P_{2}} \end{array}\right.$. Combining (4), (3) can be rewritten as

(5) $$\left\{\begin{array}{c} Q_{1}=\gamma R_{1}u\\ Q_{2}=\gamma R_{2}u \end{array}\right.$$

Equations (1), (2) and (5) completely describe the third-order dynamics of the EHSS in the HIR. Defining the state variables as $X={\left[x_{1},x_{2},x_{3}\right]}^{T}={\left[x_{p},{\dot{x}}_{p},{\ddot{x}}_{p}\right]}^{T}$, then the system dynamics can be expressed in the integral chain form as

(6) $$\left\{\begin{array}{c} {\dot{x}}_{1}=x_{2}\\ {\dot{x}}_{2}=x_{3}\\ {\dot{x}}_{3}=a_{1}x_{2}+a_{2}x_{3}+bu+d \end{array}\right.$$

where $a_{1}=\frac{1}{m}\left(-\frac{\beta_{e}A_{1}^{2}}{V_{1}}-\frac{\beta_{e}A_{2}^{2}}{V_{2}}\right)$, $a_{2}=-\frac{B}{m}$, $b=\frac{1}{m}\left(\frac{\beta_{e}A_{1}}{V_{1}}\gamma R_{1}+\frac{\beta_{e}A_{2}}{V_{2}}\gamma R_{2}\right)$ and $d=\frac{1}{m}\left(-\frac{\beta_{e}A_{1}C_{i}\left(p_{1}-p_{2}\right)}{V_{1}}-\frac{\beta_{e}A_{2}C_{i}\left(p_{1}-p_{2}\right)}{V_{2}}-{\dot{F}}_{L}\left(t,x_{p},{\dot{x}}_{p}\right)\right)$.

## 3. Controller Design

### 3.1. ESO Design

Since the hydraulic cylinders used in practice are usually only installed with displacement sensors for the detection of the cylinder rod’s position, it is difficult to obtain the state information such as the actuator’s velocity and acceleration directly through components, and the system disturbances cannot be accurately estimated and effectively suppressed. Therefore, ESO is designed to address the above issues.

The constant gain *b_n_* is used instead of the nonlinear gain *b* in system (6). Defined $\tilde{b}=b_{n}-b$ as the difference between *b_n_* and *b*. Then, (6) can be rewritten as

(7) $$\left\{\begin{array}{c} {\dot{x}}_{1}=x_{2}\\ {\dot{x}}_{2}=x_{3}\\ {\dot{x}}_{3}=b_{n}u+\delta \end{array}\right.$$

where $\delta =a_{1}x_{2}+a_{2}x_{3}+d-\tilde{b}u$ represents the lumped disturbances, which include unknowns, uncertainties, coupled dynamics and modeling errors. On the assumption that *δ* is bounded and differentiable, define *x*_4_ = *δ* as the fourth system state. Then, the state vector *X* is expressed in four-dimensional form, i.e., $X={\left[x_{1},x_{2},x_{3},x_{4}\right]}^{T}={\left[x_{p},{\dot{x}}_{p},{\ddot{x}}_{p},\delta \right]}^{T}$. The original system (7) is rewritten as

(8) $$\left\{\begin{array}{c} {\dot{x}}_{1}=x_{2}\\ {\dot{x}}_{2}=x_{3}\\ {\dot{x}}_{3}=b_{n}u+x_{4}\\ {\dot{x}}_{4}=\dot{\delta } \end{array}\right.$$

Defining the estimation vector of $X={\left[x_{1},x_{2},x_{3},x_{4}\right]}^{T}$ as $\hat{X}={\left[{\hat{x}}_{1},{\hat{x}}_{2},{\hat{x}}_{3},{\hat{x}}_{4}\right]}^{T}$, and a linear type of the ESO [36] is designed as

(9) $$\left\{\begin{array}{c} {\dot{\hat{x}}}_{1}={\hat{x}}_{2}+4\omega_{0}\left(x_{1}-{\hat{x}}_{1}\right)\\ {\dot{\hat{x}}}_{2}={\hat{x}}_{3}+6{\omega_{0}}^{2}\left(x_{1}-{\hat{x}}_{1}\right)\\ {\dot{\hat{x}}}_{3}=b_{n}u+{\hat{x}}_{4}+4{\omega_{0}}^{3}\left(x_{1}-{\hat{x}}_{1}\right)\\ {\dot{\hat{x}}}_{4}={\omega_{0}}^{4}\left(x_{1}-{\hat{x}}_{1}\right) \end{array}\right.$$

where *ω*_0_ is considered to be the observer bandwidth, *ω*_0_ > 0.

Defined $\tilde{X}=X-\hat{X}$ as the error vector, and $\eta =\Gamma \tilde{X}$ as the constructed ESO error vector. Combining (8) and (9), the dynamic equation of the observation error is given as

(10) $$\dot{\eta }=\omega_{0}A\eta +{\omega_{0}}^{-3}B\dot{\delta }$$

where $\Gamma =\left[\begin{array}{cccc} 1 & 0 & 0 & 0\\ 0 & {\omega_{0}}^{-1} & 0 & 0\\ 0 & 0 & {\omega_{0}}^{-2} & 0\\ 0 & 0 & 0 & {\omega_{0}}^{-3} \end{array}\right]$, $A=\left[\begin{array}{cccc} -4 & 1 & 0 & 0\\ -6 & 0 & 1 & 0\\ -4 & 0 & 0 & 1\\ -1 & 0 & 0 & 0 \end{array}\right]$ and $B=\left[\begin{array}{c} 0\\ 0\\ 0\\ 1 \end{array}\right]$.

The system matrix *A* of (10) is Hurwitz. Thus, there exists a symmetrical positive definite matrix *P* satisfying the following equation

(11) $$A^{T}P+PA=-2I$$

Define the Lyapunov function of the observer as $V_{o}=\frac{1}{2}\eta^{T}P\eta$, then the derivative of *V_o_* is given as

(12) $${\dot{V}}_{o}=\frac{1}{2}\left({\dot{\eta }}^{T}P\eta +\eta^{T}P\dot{\eta }\right)\;=\frac{1}{2}\left[\omega_{0}\eta^{T}\left(A^{T}P+PA\right)\eta +2{\omega_{0}}^{-3}\eta^{T}PB\dot{\delta }\right]\;\;\leq -\omega_{0}{\left\|\eta \right\|}^{2}+\frac{1}{2}{\left\|\eta \right\|}^{2}+\frac{1}{2}{\omega_{0}}^{-6}{\left\|PB\right\|}^{2}{\left|\dot{\delta }\right|}_{max}^{2}\;\;\leq -\left(\omega_{0}-\frac{1}{2}\right)\frac{1}{\lambda_{max}\left(P\right)}\eta^{T}P\eta +\frac{1}{2}{\omega_{0}}^{-6}{\left\|PB\right\|}^{2}{\left|\dot{\delta }\right|}_{max}^{2}\;=-\frac{2\omega_{0}-1}{\lambda_{max}\left(P\right)}V_{o}+\frac{1}{2}{\omega_{0}}^{-6}{\left\|PB\right\|}^{2}{\left|\dot{\delta }\right|}_{max}^{2}$$

By the comparison lemma [37], it can be inferred that the ESO in (9) is stable, while the convergence rate and observation accuracy are determined by the bandwidth *ω*_0_.

### 3.2. ESO-Based Chattering-Free TSM Controller Design

Given *x*_1*r*_ as the position reference of the cylinder rod, the tracking error is defined as

(13) $$\left\{\begin{array}{c} e_{1}=x_{1}-x_{1r}\\ e_{2}=x_{2}-{\dot{x}}_{1r}\\ e_{3}=x_{3}-{\ddot{x}}_{1r} \end{array}\right.$$

Combining the system (7), the error dynamics can be expressed as

(14) $$\left\{\begin{array}{c} {\dot{e}}_{1}=e_{2}\\ {\dot{e}}_{2}=e_{3}\\ {\dot{e}}_{3}=b_{n}u+\delta -{\dddot{x}}_{1r} \end{array}\right.$$

The chattering-free TSM manifold [19] of the same order as the system (14) can be constructed as

(15) $$s={\dot{e}}_{3}+\beta_{3}\mathrm{sgn}\left(e_{3}\right){\left|e_{3}\right|}^{\sigma_{3}}+\beta_{2}\mathrm{sgn}\left(e_{2}\right){\left|e_{2}\right|}^{\sigma_{2}}+\beta_{1}\mathrm{sgn}\left(e_{1}\right){\left|e_{1}\right|}^{\sigma_{1}}$$

where *β_i_* (*i* = 1, 2, 3) are selected such that the polynomial $p^{3}+\beta_{3}p^{2}+\beta_{2}p+\beta_{1}$ is a Hurwitz polynomial. *σ_i_* (*i* = 1, 2, 3) are given as follows [17]

(16) $$\left\{\begin{array}{c} \sigma_{i-1}=\frac{\sigma_{i}\sigma_{i+1}}{2\sigma_{i+1}-\sigma_{i}},i=2,3\\ \begin{array}{c} \sigma_{3}=\sigma \\ \sigma_{4}=1 \end{array} \end{array}\right.$$

where $\sigma \in \left(0,\;1\right)$.

The estimated states are used to replace the actual states in the above equations, and (15) can be rewritten as

(17) $$\hat{s}={\dot{\hat{e}}}_{3}+\beta_{3}\mathrm{sgn}\left({\hat{e}}_{3}\right){\left|{\hat{e}}_{3}\right|}^{\sigma_{3}}+\beta_{2}\mathrm{sgn}\left({\hat{e}}_{2}\right){\left|{\hat{e}}_{2}\right|}^{\sigma_{2}}+\beta_{1}\mathrm{sgn}\left({\hat{e}}_{1}\right){\left|{\hat{e}}_{1}\right|}^{\sigma_{1}}$$

where $\left\{\begin{array}{c} {\hat{e}}_{1}={\hat{x}}_{1}-x_{1r}\\ {\hat{e}}_{2}={\hat{x}}_{2}-{\dot{x}}_{1r}\\ {\hat{e}}_{3}={\hat{x}}_{3}-{\ddot{x}}_{1r} \end{array}\right.$ and ${\dot{\hat{e}}}_{3}={\dot{\hat{x}}}_{3}-{\dddot{x}}_{1r}=b_{n}u+{\hat{x}}_{4}+4{\omega_{0}}^{3}\left(x_{1}-{\hat{x}}_{1}\right)-{\dddot{x}}_{1r}$.

The control law is designed as

(18) $$u={b_{n}}^{-1}\left(\begin{array}{c} u_{eq}+u_{n} \end{array}\right)$$

Let *s* = 0 and substituting the estimated disturbance ${\hat{x}}_{4}$ into the control law for feedforward compensation, and the equivalent control law *u_eq_* is given as follows

(19) $$u_{eq}={\dddot{x}}_{1r}-{\hat{x}}_{4}-\beta_{3}\mathrm{sgn}\left({\hat{e}}_{3}\right){\left|{\hat{e}}_{3}\right|}^{\sigma_{3}}-\beta_{2}\mathrm{sgn}\left({\hat{e}}_{2}\right){\left|{\hat{e}}_{2}\right|}^{\sigma_{2}}-\beta_{1}\mathrm{sgn}\left({\hat{e}}_{1}\right){\left|{\hat{e}}_{1}\right|}^{\sigma_{1}}$$

The *u_n_* is obtained by a low-pass filter as follows

(20) $${\dot{u}}_{n}+Tu_{n}=v$$

where *T* ≥ 0 is a small constant.

The composite reaching law is chosen as

(21) $$v=-K\tanh\left(\frac{\hat{s}}{\varepsilon }\right)-k_{g}\hat{s}$$

where $\varepsilon \in \left(0,\;1\right)$ and

(22) $$K=k_{d}+k_{T}$$

The Laplace transfer function of the filter (20) is

(23) $$\frac{u_{n}\left(s\right)}{v\left(s\right)}=\frac{1}{s+T}$$

If *T* = 0, the transfer function can be written as

(24) $$\frac{u_{n}\left(s\right)}{v\left(s\right)}=\frac{1}{s}$$

The system (14) is chattering-free if the control output *u* is designed as (18). The reason is that the switch function in (21) is non-smooth, but *u_n_*(*t*) in (18) is smoothed by the filter (20).

Define $\tilde{\omega }$ as

(25) $$\tilde{\omega }=\left[\beta_{3}\mathrm{sgn}\left(e_{3}\right){\left|e_{3}\right|}^{\sigma_{3}}+\beta_{2}\mathrm{sgn}\left(e_{2}\right){\left|e_{2}\right|}^{\sigma_{2}}+\beta_{1}\mathrm{sgn}\left(e_{1}\right){\left|e_{1}\right|}^{\sigma_{1}}\right]\;-\left[\beta_{3}\mathrm{sgn}\left({\hat{e}}_{3}\right){\left|{\hat{e}}_{3}\right|}^{\sigma_{3}}+\beta_{2}\mathrm{sgn}\left({\hat{e}}_{2}\right){\left|{\hat{e}}_{2}\right|}^{\sigma_{2}}+\beta_{1}\mathrm{sgn}\left({\hat{e}}_{1}\right){\left|{\hat{e}}_{1}\right|}^{\sigma_{1}}\right]$$

From (14), (18), (19) and (25), (15) can be simplified as follows

(26) $$s=u_{n}+\tilde{\delta }+\tilde{\omega }$$

where $\tilde{\delta }={\tilde{x}}_{4}=\delta -{\hat{x}}_{4}$.

Derivation of (26) can be given as

(27) $$\dot{s}={\dot{u}}_{n}+\dot{\tilde{\delta }}+\dot{\tilde{\omega }}$$

Defining the Lyapunov function of the controller as $V_{c}=\frac{1}{2}s^{2}$, combining (20), (21), (26) and (27), the derivative of *V_c_* can be given as

(28) $${\dot{V}}_{c}=s\dot{s}=s\left(\dot{\tilde{\delta }}+\dot{\tilde{\omega }}+{\dot{u}}_{n}\right)\;=s\left(\dot{\tilde{\delta }}+\dot{\tilde{\omega }}+{\dot{u}}_{n}+Tu_{n}-Tu_{n}\right)\;=s\left(\dot{\tilde{\delta }}+\dot{\tilde{\omega }}+v-T\left(s-\tilde{\delta }-\tilde{\omega }\right)\right)\;\;\;\;\;\;\;\;\;\;=s\left(\dot{\tilde{\delta }}+\dot{\tilde{\omega }}-Ts+T\tilde{\delta }+T\tilde{\omega }\right)-s\cdot K\tanh\left(\frac{\hat{s}}{\varepsilon }\right)-s\cdot k_{g}\hat{s}$$

Defined $\tilde{\tau }$ and $\tilde{s}$ as

(29) $$\left\{\begin{array}{c} \tilde{\tau }=\tanh\left(\frac{s}{\varepsilon }\right)-\tanh\left(\frac{\hat{s}}{\varepsilon }\right)\\ \tilde{s}=s-\hat{s} \end{array}\right.$$

Then, substituting (29) into (28) yields

(30) $${\dot{V}}_{c}=s\left(\dot{\tilde{\delta }}+\dot{\tilde{\omega }}-Ts+T\tilde{\delta }+T\tilde{\omega }\right)-s\cdot K\tanh\left(\frac{s}{\varepsilon }\right)-k_{g}s^{2}+s\cdot K\tilde{\tau }+s\cdot k_{g}\tilde{s}$$

Applying the claim in [38], the following can be obtained

(31) $$-Ks\tanh\left(\frac{s}{\varepsilon }\right)⩽-K\left|s\right|+\mu K\varepsilon$$

where μ is a constant, and μ=0.2875.

To ensure that the system (14) is stable, the parameters in (21) and (22) are chosen as follows

(32) $$\left\{\begin{array}{c} k_{d}\geq \left|\dot{\tilde{\delta }}+\dot{\tilde{\omega }}\right|\\ k_{T}\geq \left|T\tilde{\delta }+T\tilde{\omega }+k_{g}\tilde{s}\right|\\ k_{g}>\frac{1}{2}-T \end{array}\right.$$

Substituting (22) and (31) into (30) yields

(33) $${\dot{V}}_{c}\leq s\left(\dot{\tilde{\delta }}+\dot{\tilde{\omega }}-Ts+T\tilde{\delta }+T\tilde{\omega }\right)-\left(k_{d}+k_{T}\right)\left|s\right|-k_{g}s^{2}+s\cdot K\tilde{\tau }+s\cdot k_{g}\tilde{s}+\mu K\varepsilon \;=\left[\left(\dot{\tilde{\delta }}+\dot{\tilde{\omega }}\right)s-k_{d}\left|s\right|\right]+\left[\left(T\tilde{\delta }+T\tilde{\omega }+k_{g}\tilde{s}\right)s-k_{T}\left|s\right|\right]-\left(T+k_{g}\right)s^{2}+s\cdot K\tilde{\tau }+\mu K\varepsilon \;\;\leq -\left(T+k_{g}\right)s^{2}+\frac{1}{2}s^{2}+\frac{1}{2}{\left|K\tilde{\tau }\right|}_{max}^{2}+\mu K\varepsilon \;=-\left(2T+2k_{g}-1\right)V_{c}+\frac{1}{2}{\left|K\tilde{\tau }\right|}_{max}^{2}+\mu K\varepsilon$$

Considering the closed-loop system formed by the observer and the controller together, the Lyapunov function is expressed as $V=V_{o}+V_{c}$. From (12) and (33), the derivative of *V* can be written as

(34) $$\dot{V}\leq -\phi V+\xi$$

where $\phi =min\left\{\frac{2\omega_{0}-1}{\lambda_{max}\left(P\right)},2T+2k_{g}-1\right\}$ and $\xi =\frac{1}{2}{\omega_{0}}^{-6}{\left\|PB\right\|}^{2}{\left|\dot{\delta }\right|}_{max}^{2}+\frac{1}{2}{\left|K\tilde{\tau }\right|}_{max}^{2}+\mu K\varepsilon$.

Applying the comparison lemma [37] for inequality (34) yields

(35) $$V(t)\leq V(0)\exp(-\phi t)+\frac{\xi }{\phi }\left[1-\exp(-\phi t)\right]$$

Thus, the function *V* is bounded by $\frac{\xi }{\phi }$, which means the closed-loop system is stable. If *K*, *k*g and *ω*_0_ are sufficiently large, while *ε* is sufficiently small, the function *V* tends to be zero in finite-time.

**Figure 3:**
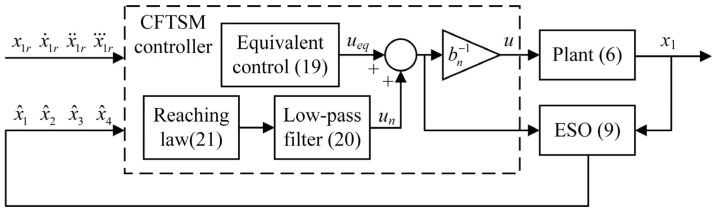
Control block diagram of the system.

Equations (9) and (18)–(22) show the complete structure of the proposed ESO-CFTSM in Figure 3, and the parameters to be tuned are as follows: (*b_n_*, ω_0_, *β*_1_, *β*_2_, *β*_3_, *σ*, *T*, *K*, *k_g_*, *ε*). First of all, the ω_0_ and *b_n_* in the ESO (9) should be set. The ω_0_ can be tuned with a simple proportional controller to obtain the desired estimation performance. Furthermore, as the $\tilde{b}$ has already been observed and compensated for within the lumped disturbance *x*_4_, the accuracy of the *b_n_* is not a primary concern. However, it should be noted that the *b_n_* determines the control gain in (18). Regarding the parameters *β*_1_, *β*_2_, *β*_3_, and *σ* in (15), a smaller fractional power *σ* can provide the system with a faster convergence rate near the equilibrium point. Once *σ* has been selected, *β*_1_, *β*_2_ and *β*_3_ can be tuned with the pole placement method to achieve the desired system dynamics. In (21), the *ε* in the nonlinear term is set to a small value to reasonably approximate the sign function, and the nonlinear gain *K* enhances control robustness. The *k_g_* in the linear term ensures fast convergence towards the sliding mode when the function *s* (15) is large. A sufficiently small T can filter out all high-frequency noise caused by the switching function.

It is worth pointing out that the parameters *b_n_*, *T*, *K*, *k_g_* and *ε* can be easily set. At the same time, a *σ* closer to 0, a larger observer bandwidth *ω*_0_ and larger gains *β_i_* (*i* = 1, 2, 3) can achieve the faster response and the higher accuracy, but unreasonable values will cause the system to over-respond and amplify high-frequency noise. Therefore, in practical applications, the parameters tuning of both the observer and controller are subject to factors such as measurement noise, the specified sampling rate, and system dynamics. A trade-off between different parameters must be made to achieve optimal control performance.

## 4. Simulations Verification

### 4.1. Simulation Setup

**Figure 4:**
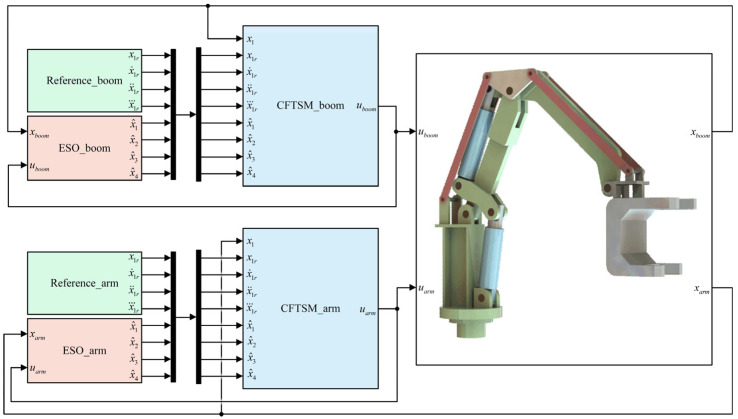
Simulation model with MATLAB R2023b/Simulink.

A simulation model of the 2-DOF HIR with coupled dynamics was setup by using MATLAB R2023b/Simulink. The developed simulation model is shown in Figure 4. The two cylinders of the HIR have the same piston and rod diameters: 150 mm and 75 mm, and their strokes are 300 mm and 420 mm, respectively. The translational friction of the hydraulic cylinders is modeled using the Stribeck friction model. Two identical servo valves with second-order dynamics were used to control the actuators. The total mass of the pliers and load is 2000 kg, and the damping coefficient of revolute joints was set to 300 N·m/(deg/s). The main simulation parameters are shown in Table 1.

**Figure 5:**
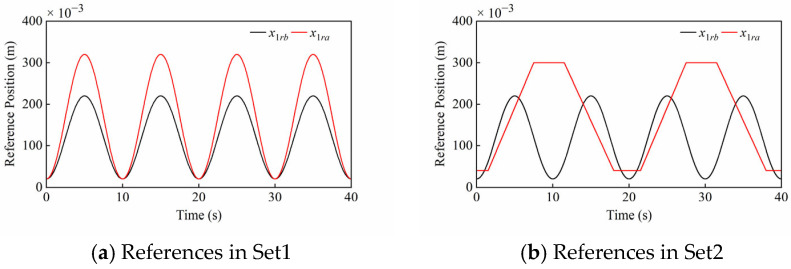
Motion references of boom cylinder (*x*_1*rb*_) and arm cylinder (*x*_1*ra*_) in Set1 and Set2.

As shown in Figure 5, two sets of compound motion tracking simulations were performed to verify the effectiveness of the proposed controller. Define *x*_1*rb*_ and *x*_1*ra*_ as the position references of the boom cylinder and arm cylinder in the HIR, respectively.

Set1: Compound motion tracking with different sinusoidal references, which can verify the robust performance against the periodic disturbance. Specifically, $x_{1rb}$ is the described sinusoidal reference: $x_{1rb}=100\sin\left(0.2\pi t-0.5\pi \right)+120$ mm; $x_{1ra}$ is the described sinusoidal reference: $x_{1ra}=150\sin\left(0.2\pi t-0.5\pi \right)+170$ mm.

Set2: Compound motion tracking with sinusoidal and trapezoidal references, which can verify the robust performance against the composite disturbance. Specifically, $x_{1rb}$ is the described sinusoidal reference: $x_{1rb}=100\sin\left(0.2\pi t-0.5\pi \right)+120$ mm; $x_{1ra}$ is the trapezoidal reference with lower and upper bounds of 40 mm and 300 mm, where extension and retraction times of the arm cylinder are 6 s and 6.5 s, respectively.

### 4.2. Controllers for Comparison

To verify the superiority of the proposed ESO-CFTSM, the other three control methods, including the ESO-based linear state feedback (ESO-LSF), the ESO-based nonlinear state feedback (ESO-NSF), and the ESO-based chattering-free LSM (ESO-CFLSM), were performed as comparison schemes. The structure of the ESOs in all controllers is the same as the ESO in (9). In addition, to ensure a fair comparison of the control performance, the ESO parameters were set the same in all controllers, including *b_n_*, and *ω*_0_. For simplicity, the writing of ESOs is omitted in the following control laws.

Controller *C*1: *C*1 stands for the ESO-LSF controller. This is a linear state feedback controller, which includes the disturbance compensation. The control law is given as follows

(36) $$u={b_{n}}^{-1}\left({\dddot{x}}_{1r}-{\hat{x}}_{4}-\alpha_{3}{\hat{e}}_{3}-\alpha_{2}{\hat{e}}_{2}-\alpha_{1}{\hat{e}}_{1}\right)$$

where *α_i_* (*i* = 1, 2, 3) are the linear state feedback gains.

Controller *C*2: *C*2 stands for the ESO-NSF controller. This is a nonlinear state feedback controller similar to the proposed ESO-CFTSM but without the sliding mode, which includes the disturbance compensation. The control law is given as follows

(37) $$u={b_{n}}^{-1}\left({\dddot{x}}_{1r}-{\hat{x}}_{4}-\beta_{3}\mathrm{sgn}\left({\hat{e}}_{3}\right){\left|{\hat{e}}_{3}\right|}^{\sigma_{3}}-\beta_{2}\mathrm{sgn}\left({\hat{e}}_{2}\right){\left|{\hat{e}}_{2}\right|}^{\sigma_{2}}-\beta_{1}\mathrm{sgn}\left({\hat{e}}_{1}\right){\left|{\hat{e}}_{1}\right|}^{\sigma_{1}}\right)$$

where all symbols are defined the same as the proposed ESO-CFTSM.

Controller *C*3: *C*3 stands for the ESO-CFLSM controller. This is a chattering-free linear sliding-mode controller similar to the proposed ESO-CFTSM, but with a linear feedback control law. The control law is given as follows

(38) $$\left\{\begin{array}{c} \hat{s}={\dot{\hat{e}}}_{3}+\alpha_{3}{\hat{e}}_{3}+\alpha_{2}{\hat{e}}_{2}+\alpha_{1}{\hat{e}}_{1}\\ u={b_{n}}^{-1}\left(\begin{array}{c} u_{eq}+u_{n} \end{array}\right)\\ u_{eq}={\dddot{x}}_{1r}-{\hat{x}}_{4}-\alpha_{3}{\hat{e}}_{3}-\alpha_{2}{\hat{e}}_{2}-\alpha_{1}{\hat{e}}_{1}\\ {\dot{u}}_{n}+Tu_{n}=v\\ v=-K\tanh\left(\frac{\hat{s}}{\varepsilon }\right)-k_{g}\hat{s} \end{array}\right.$$

where parameters *α_i_* (*i* = 1, 2, 3) are defined in the ESO-LSF, and other symbols are defined the same as the proposed ESO-CFTSM.

Controller *C*4: *C*4 stands for the proposed ESO-CFTSM controller. The proposed ESO-CFTSM control law in Section 3 can be summarized as the following equation

(39) $$\left\{\begin{array}{c} \hat{s}={\dot{\hat{e}}}_{3}+\beta_{3}sgn\left({\hat{e}}_{3}\right){\left|{\hat{e}}_{3}\right|}^{\sigma_{3}}+\beta_{2}sgn\left({\hat{e}}_{2}\right){\left|{\hat{e}}_{2}\right|}^{\sigma_{2}}+\beta_{1}sgn\left({\hat{e}}_{1}\right){\left|{\hat{e}}_{1}\right|}^{\sigma_{1}}\\ u={b_{n}}^{-1}\left(\begin{array}{c} u_{eq}+u_{n} \end{array}\right)\\ u_{eq}={\dddot{x}}_{1r}-{\hat{x}}_{4}-\beta_{3}sgn\left({\hat{e}}_{3}\right){\left|{\hat{e}}_{3}\right|}^{\sigma_{3}}-\beta_{2}sgn\left({\hat{e}}_{2}\right){\left|{\hat{e}}_{2}\right|}^{\sigma_{2}}-\beta_{1}sgn\left({\hat{e}}_{1}\right){\left|{\hat{e}}_{1}\right|}^{\sigma_{1}}\\ {\dot{u}}_{n}+Tu_{n}=v\\ v=-K\tanh\left(\frac{\hat{s}}{\varepsilon }\right)-k_{g}\hat{s} \end{array}\right.$$

where all parameters for the ESO-CFTSM were tuned according to the simulation results and the tuning method.

The control parameters of the controllers are shown in Table 2.

It is worth noting that the ESO bandwidth ω_0_ of the arm cylinder in Set2 was set to 300 in order to reduce observation errors under composite disturbance. Moreover, compared to the nonlinear feedback gains *β_i_* (*i* = 1, 2, 3) of *C*2 and *C*4, the linear feedback gains *α_i_* (*i* = 1, 2, 3) of *C*1 and *C*3 were set to larger values in order to obtain the desired control performance in both sets of simulations, which proves the advantage of the nonlinear feedback control law.

During the simulations, it was found that the dynamic performance was unsatisfactory if the parameters *β_i_* (*i* = 1, 2, 3) of *C*2 in Set1 and the parameter *K* of *C*3 in Set2 were selected the same as *C*4. Therefore, more reasonable *β_i_* (*i* = 1, 2, 3) and *K* were set in parentheses of Table 2. Specifically, the *C*2 parameters of the boom cylinder in Set1 were tuned as *β*_1_ = 512, *β*_2_ = 192 and *β*_3_ = 24, the *C*2 parameters of the arm cylinder in Set1 were tuned as *β*_1_ = 800, *β*_2_ = 260 and *β*_3_ = 28, and the *C*3 parameter of the arm cylinder in Set2 was tuned as *K* = 500. This does not lose the fairness of the comparison, because it is demonstrated that the proposed ESO-CFTSM has lower control gains than other controllers.

To quantitatively evaluate the controller performance, the following three sets of performance indexes are defined, i.e., the absolute peak error (APE), the integral absolute error (IAE) and the integral time-weighted absolute error (ITAE).

(40) $$\left\{\begin{array}{c} APE=\underset{0<t\leq t_{1}}{max}\left\{\left|e_{1}\right|\right\}\\ IAE=\int_{0}^{t_{1}}\left|e_{1}\right|dt\\ ITAE=\int_{0}^{t_{1}}t\left|e_{1}\right|dt \end{array}\right.$$

where $e_{1}=x_{1}-x_{1r}$ represents the position tracking error of cylinders.

### 4.3. Results and Analysis of Controller Performances

#### 4.3.1. Tracking Accuracy

**Figure 6:**
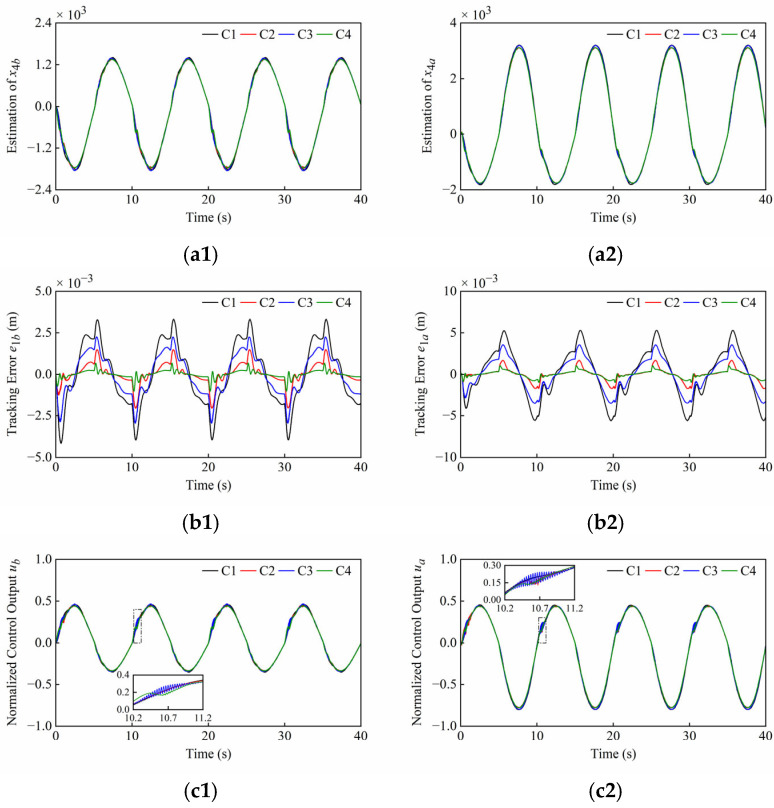
Simulation results of (**a1**–**c1**) boom cylinder and (**a2**–**c2**) arm cylinder under normal operating conditions of Set1. From top to bottom: disturbance estimation *x*_4_; tracking error *e*_1_; normalized control output *u*.

**Figure 7:**
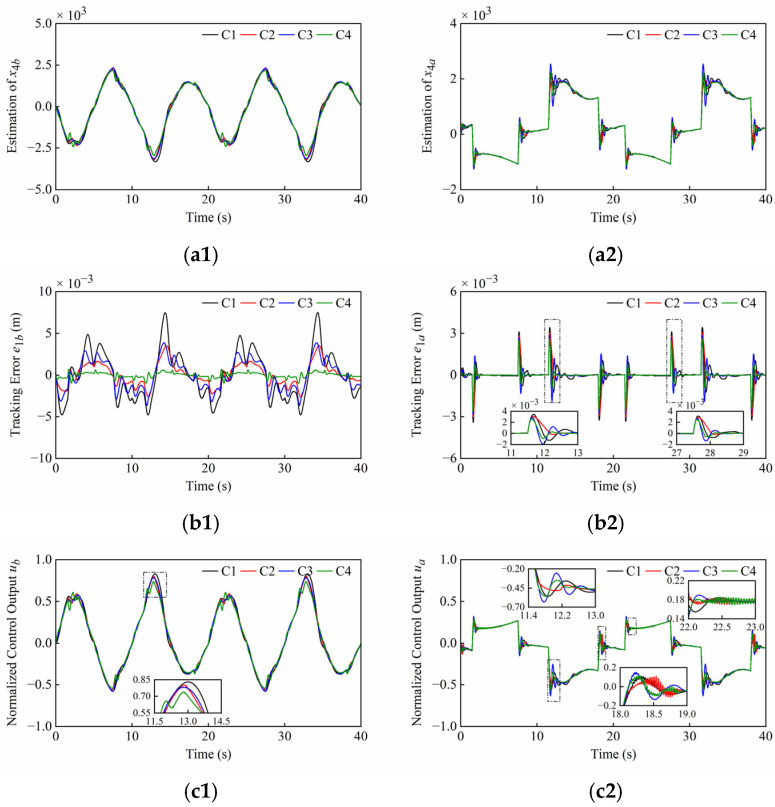
Simulation results of (**a1**–**c1**) boom cylinder and (**a2**–**c2**) arm cylinder under normal operating conditions of Set2. From top to bottom: Disturbance estimation *x*_4_; Tracking error *e*_1_; Normalized control output *u*.

Firstly, the tracking accuracy of the plant under normal operating conditions is discussed. The system and controller parameters are presented in Table 1 and Table 2. The simulation results under normal operating conditions of Set1 and Set2 are shown in Figure 6 and Figure 7, noting that the different scales were applied in the plots. These simulation results include disturbance estimation *x*_4_ of the ESO, tracking error *e*_1_, and normalized control output *u*. The quantitative performance indexes of the different controllers are given in Table 3 and Table 4.

From Figure 6(a1,a2) and Figure 7(a1,a2), it can be seen that the ESOs have desired estimation results for lumped disturbances of two cylinders in both sets of simulations. Moreover, it can be found that the amplitudes of the control outputs of four controllers are similar and the singularities are avoided in Figure 6(c1,c2) and Figure 7(c1,c2).

As shown in Figure 6(b1,b2), *C*2 and *C*4, both with nonlinear state feedback laws, have faster convergence speeds compared to *C*1 and *C*3, both with linear state feedback laws. Meanwhile, the steady state errors of *C*3 and *C*4 with the sliding mode are smaller than those of *C*1 and *C*2 without the sliding mode, respectively. As illustrated in the scaling section of Figure 6(c1,c2), the control outputs of *C*1, *C*2, and *C*3 oscillated slightly due to the friction when both cylinders started to extend, but this phenomenon did not occur in the system of *C*4 due to the rapid response.

As shown in Figure 7(b1), it is difficult to maintain high tracking accuracy of the boom cylinder with *C*1, *C*2 and *C*3 when the direction changes, but the tracking error of the boom cylinder with *C*4 is very small. From Figure 7(a1,c1), the peaks of the disturbance estimation and control output for *C*1, *C*2, and *C*3 are relatively high. In contrast, *C*4 reacts relatively slightly to direction changes. Thanks to faster convergence rate of *C*4, the boom cylinder enables smooth directional changes. As seen in the scaling section of Figure 7(b2), the system overshoots occur in all controllers, and *C*4 converges significantly faster than the compared controllers. From Figure 7(a2,c2), this phenomenon can be explained as follows: At the junctions of the trapezoidal reference, step changes in the arm cylinder velocity led to corresponding step changes in the lumped disturbances including the friction terms. This progression further led to overshoots on the disturbance estimation and control outputs, and finally to overshoots on the position tracking. As illustrated in the scaling section of Figure 7(c2), the weak chattering that is allowed on control outputs of C2 and C4 occurs during the uniform extension stage of the arm cylinder. This is due to the terms with fractional powers in their control laws. Although these terms improve the convergence rate, they result in unsmooth control outputs near equilibrium points.

#### 4.3.2. Robustness to Load Disturbances

**Figure 8:**
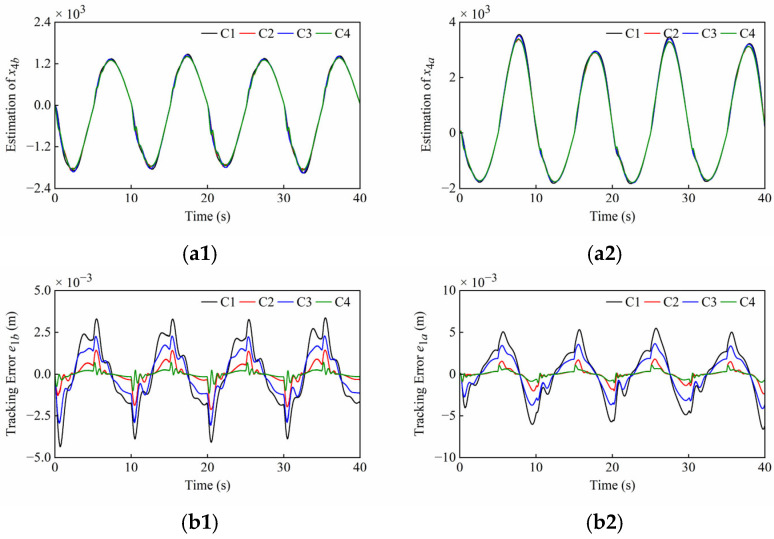

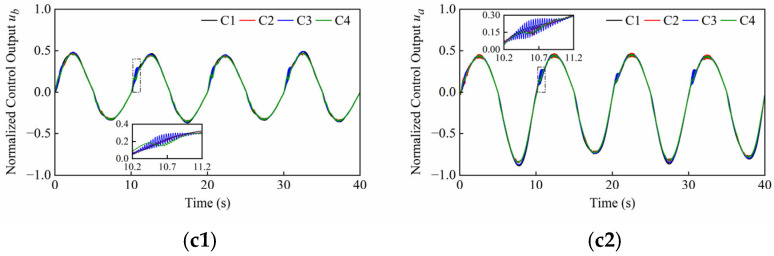
Simulation results of (**a1**–**c1**) boom cylinder and (**a2**–**c2**) arm cylinder under load disturbances of Set1. From top to bottom: disturbance estimation *x*_4_; tracking error *e*_1_; normalized control output *u*.

**Figure 9:**
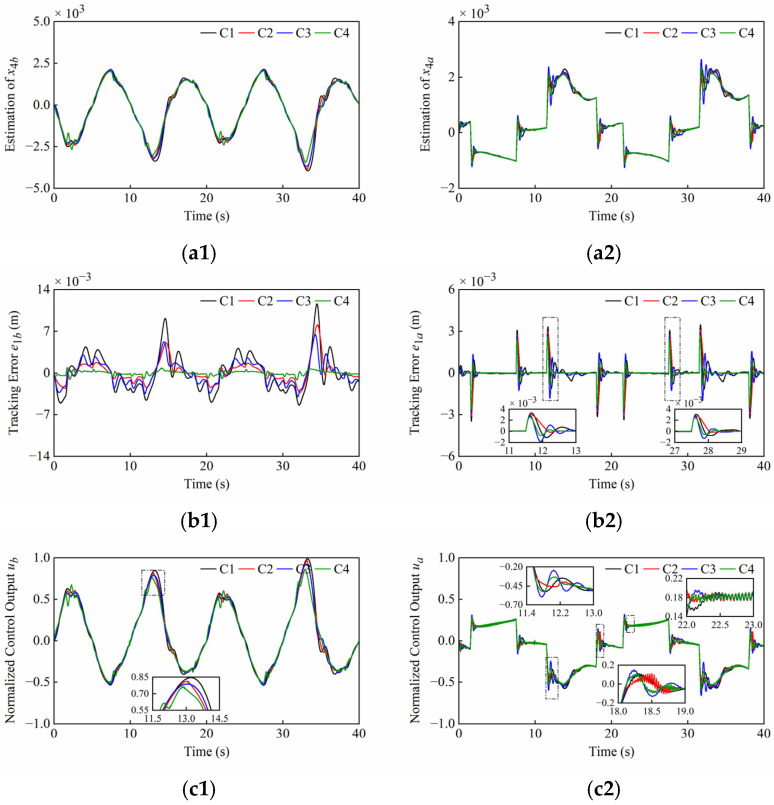
Simulation results of (**a1**–**c1**) boom cylinder and (**a2**–**c2**) arm cylinder under load disturbances of Set2. From top to bottom: disturbance estimation *x*_4_; tracking error *e*_1_; normalized control output *u*.

The plant may be subject to the external disturbances caused by unknown loads during operation. To evaluate the controller’s robustness to load disturbances, the disturbances containing both high-frequency and low-frequency components were simultaneously applied to the boom and arm cylinders. *F_db_* and *F_da_* are defined as the load disturbances of the boom cylinder and arm cylinder in the HIR, respectively. Due to their different positions, the cylinders are subject to opposing external forces, which are expressed, respectively, as $F_{db}=20,000\sin\left(t\right)+5000\sin\left(40\pi t\right)$ N and $F_{da}=-F_{db}$. The system and controller parameters are presented in Table 1 and Table 2. The simulation results under load disturbances of Set1 and Set2 are shown in Figure 8 and Figure 9. The quantitative performance indexes of the different controllers are given in Table 5 and Table 6.

From Figure 8 and Figure 9, the ESOs can still adequately estimate the lumped disturbances, and the control output can be adjusted in real-time to counteract load disturbances. The introduction of load disturbances causes the amplitude and dynamics of the disturbance estimation to behave in a different way. The high-frequency components within load disturbances cause controller chattering at specific conditions, yet *C*4 still performs satisfactorily without chattering in Set 1. The error of all controllers has increased, but this is still at a low level. *C*4 still has faster convergence and higher control accuracy. Compared to Figure 7(c1), the amplitude difference between the controller’s outputs in Figure 9(c1) has increased due to load disturbances. Unfortunately, the *C*1 control output has approached saturation, which is a phenomenon that can cause control divergence and endanger equipment operation. However, *C*4 can effectively withstand load disturbances of this scale, thereby safeguarding plant operation.

#### 4.3.3. Robustness to Parametric Uncertainties

**Figure 10:**
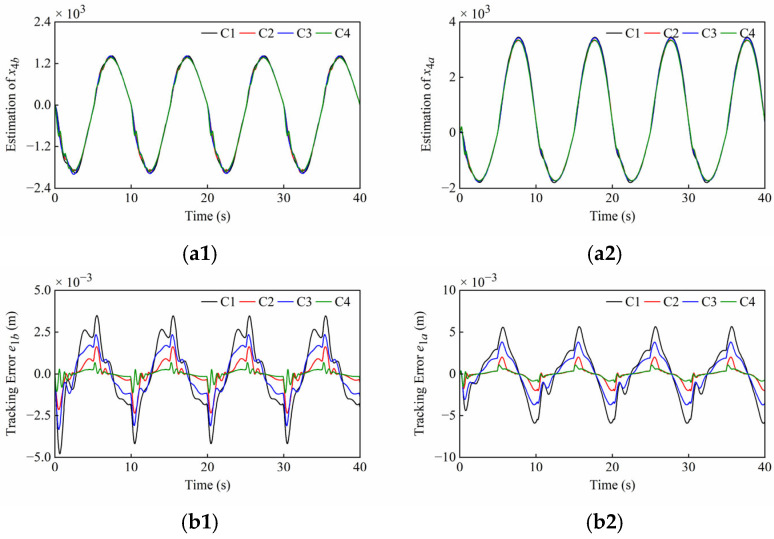

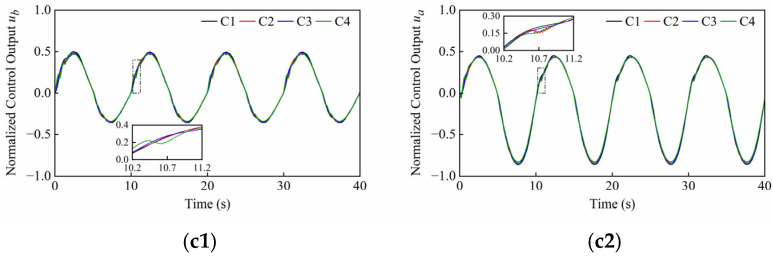
Simulation results of (**a1**–**c1**) boom cylinder and (**a2**–**c2**) arm cylinder under parametric uncertainties of Set1. From top to bottom: reference position *x*_1*r*_ and disturbance estimation *x*_4_; tracking error *e*_1_; normalized control output *u*.

**Figure 11:**
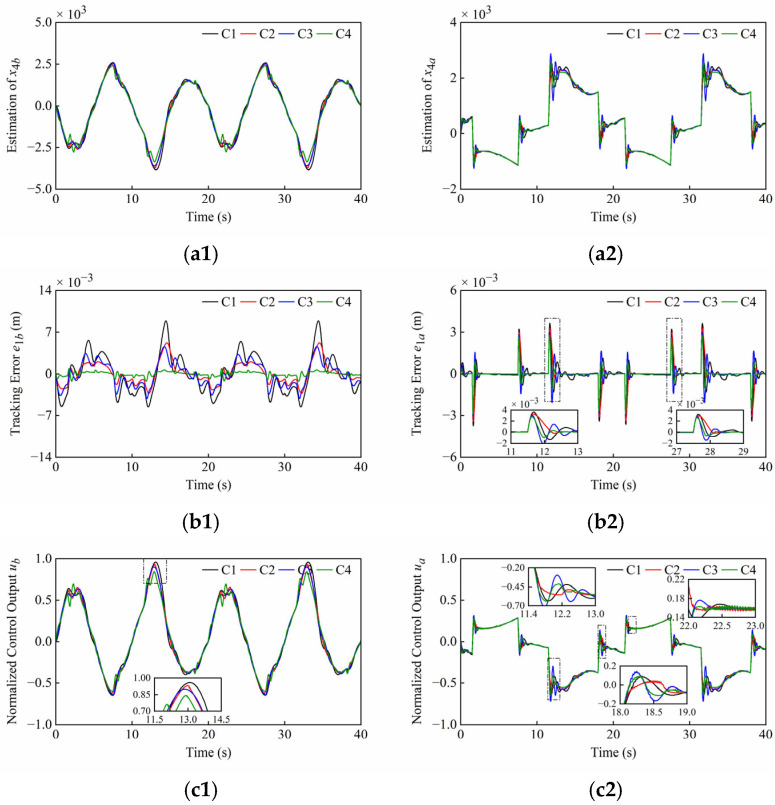
Simulation results of (**a1**–**c1**) boom cylinder and (**a2**–**c2**) arm cylinder under parametric uncertainties of Set2. From top to bottom: Disturbance estimation *x*_4_; Tracking error *e*_1_; Normalized control output *u*.

Some hydraulic parameters (*β_e_*, *C_i_*, *C_d_*, *w*, *ρ*) have parametric uncertainties due to seal wear, oil temperature change, and unknown nonlinearities of valves, etc. To demonstrate the robustness of parametric uncertainties, the internal leakage coefficient *C_i_* was set to 1.2 L/min/MPa, which differs from the previous configuration. The remaining system and controller parameters are presented in Table 1 and Table 2. The simulation results under parametric uncertainties of Set1 and Set2 are shown in Figure 10 and Figure 11. The quantitative performance indexes of the different controllers are given in Table 7 and Table 8.

From Figure 10 and Figure 11, when the internal leakage coefficient changes, the lumped disturbance amplitude changes while the control error remains low. The control output then increases to compensate for the leakage flow. It can be found that the chattering phenomenon has been eliminated in Figure 10(c1,c2), and both the amplitude and frequency of the chattering near the equilibrium point have been reduced in Figure 11(c2). The reason is that a moderate increase in internal leakage enhances the hydraulic damping ratio, thereby improving system performance and reducing oscillations. However, excessive internal leakage can increase power losses, reduce hydraulic stiffness and control gain, and may cause the plant to stop operating. From Figure 11(c1), there is still the issue that the *C*1 control output is close to the saturation state, but the *C*4 control output amplitude is relatively smaller, which can effectively cope with parametric uncertainties.

To sum up, the proposed ESO-CFTSM is nonsingular and chattering-free, and the excellent tracking performance is achieved without being affected by lumped disturbances. Based on the above analysis, the proposed ESO-CFTSM is superior to the compared controllers.

## 5. Conclusions

In this paper, an ESO-based chattering-free TSM controller is proposed for the EHSS of the hydraulic manipulator. This controller considers all uncertainties and unknowns in the system dynamics as the lumped disturbances. The estimation of the EHSS states and disturbances is obtained by the ESO, and the control accuracy is improved by the feedforward compensation of disturbances. Since the TSM manifold in the proposed controller has the same order as the system, the direct differentiation of terms with fractional powers is avoided, and the singularity is ultimately eliminated. Additionally, the hyperbolic tangent function is used in the composite reaching law, and a low-pass filter is introduced to realize chattering-free control. Simulation results verify the efficiency and superiority of the proposed control method.

## Figures and Tables

**Table 1:** Simulation parameters.

Parameter	Value	Parameter	Value
Coulomb friction	2000 N	Breakaway friction	5000 N
Viscous friction	2500 N·s/m	Supply pressure	21 MPa
Internal leakage	0.6 L/min/MPa	Maximum supply flow rate	200 L/min
Valves damping ratio	0.8	Valves natural frequency	80 Hz

**Table 2:** Control parameters used in simulations.

	Set 1		Set 2	
	Boom Cylinder	Arm Cylinder	Boom Cylinder	Arm Cylinder
*b_n_*	100	100	100	100
*ω*_0_	100	100	100	300
*α*_1_	48,000	48,000	48,000	48,000
*α*_2_	4000	4000	4000	4000
*α*_3_	110	110	110	110
*β*_1_	300 (512)	300 (800)	1000	800
*β*_2_	140 (192)	140 (260)	500	600
*β*_3_	21 (24)	21 (28)	20	100
*σ*	1/2	1/2	3/5	3/5
*T*	0.01	0.01	0.01	0.01
*K*	500	500	500	50 (500)
*k_g_*	50	50	50	10
*ε*	0.1	0.1	0.1	0.1

**Table 3:** Performance indexes under normal operating conditions of Set1.

	Boom Cylinder			Arm Cylinder		
	SAPE	IAE	ITAE	SAPE	IAE	ITAE
*C*1	4.16 × 10^−3^	6.55 × 10^−2^	12.9 × 10^−1^	5.58 × 10^−3^	10.1 × 10^−2^	20.9 × 10^−1^
*C*2	2.04 × 10^−3^	1.71 × 10^−2^	3.37 × 10^−1^	1.71 × 10^−3^	1.82 × 10^−2^	3.93 × 10^−1^
*C*3	2.94 × 10^−3^	4.42 × 10^−2^	8.66 × 10^−1^	3.55 × 10^−3^	6.75 × 10^−2^	13.9 × 10^−1^
*C*4	1.01 × 10^−3^	0.63 × 10^−2^	1.23 × 10^−1^	1.03 × 10^−3^	1.24 × 10^−2^	2.60 × 10^−1^

**Table 4:** Performance indexes under normal operating conditions of Set2.

	Boom Cylinder			Arm Cylinder		
	SAPE	IAE	ITAE	SAPE	IAE	ITAE
*C*1	7.49 × 10^−3^	9.53 × 10^−2^	18.8 × 10^−1^	3.43 × 10^−3^	11.4 × 10^−3^	2.33 × 10^−1^
*C*2	3.52 × 10^−3^	4.34 × 10^−2^	8.48 × 10^−1^	3.06 × 10^−3^	8.68 × 10^−3^	1.76 × 10^−1^
*C*3	3.88 × 10^−3^	6.35 × 10^−2^	12.4 × 10^−1^	2.87 × 10^−3^	8.05 × 10^−3^	1.65 × 10^−1^
*C*4	0.99 × 10^−3^	1.04 × 10^−2^	1.96 × 10^−1^	2.62 × 10^−3^	5.46 × 10^−3^	1.10 × 10^−1^

**Table 5:** Performance indexes under load disturbances of Set1.

	Boom Cylinder			Arm Cylinder		
	SAPE	IAE	ITAE	SAPE	IAE	ITAE
*C*1	4.35 × 10^−3^	6.63 × 10^−2^	13.0 × 10^−1^	6.61 × 10^−3^	10.3 × 10^−2^	21.2 × 10^−1^
*C*2	2.13 × 10^−3^	1.70 × 10^−2^	3.35 × 10^−1^	2.38 × 10^−3^	1.89 × 10^−2^	4.03 × 10^−1^
*C*3	3.07 × 10^−3^	4.46 × 10^−2^	8.76 × 10^−1^	4.12 × 10^−3^	6.83 × 10^−2^	14.1 × 10^−1^
*C*4	1.14 × 10^−3^	0.65 × 10^−2^	1.27 × 10^−1^	1.06 × 10^−3^	1.27 × 10^−2^	2.64 × 10^−1^

**Table 6:** Performance indexes under load disturbances of Set2.

	Boom Cylinder			Arm Cylinder		
	SAPE	IAE	ITAE	SAPE	IAE	ITAE
*C*1	11.6 × 10^−3^	10.1 × 10^−2^	20.5 × 10^−1^	3.47 × 10^−3^	11.9 × 10^−3^	2.48 × 10^−1^
*C*2	8.07 × 10^−3^	4.98 × 10^−2^	10.4 × 10^−1^	3.11 × 10^−3^	8.64 × 10^−3^	1.77 × 10^−1^
*C*3	6.44 × 10^−3^	6.46 × 10^−2^	12.9 × 10^−1^	2.90 × 10^−3^	8.44 × 10^−3^	1.78 × 10^−1^
*C*4	1.14 × 10^−3^	1.08 × 10^−2^	2.06 × 10^−1^	2.70 × 10^−3^	5.54 × 10^−3^	1.12 × 10^−1^

**Table 7:** Performance indexes under parametric uncertainties of Set1.

	Boom Cylinder			Arm Cylinder		
	SAPE	IAE	ITAE	SAPE	IAE	ITAE
*C*1	4.79 × 10^−3^	6.85 × 10^−2^	13.4 × 10^−1^	5.92 × 10^−3^	10.6 × 10^−2^	21.9 × 10^−1^
*C*2	2.35 × 10^−3^	2.10 × 10^−2^	3.97 × 10^−1^	2.02 × 10^−3^	2.16 × 10^−2^	4.52 × 10^−1^
*C*3	3.33 × 10^−3^	4.66 × 10^−2^	9.10 × 10^−1^	3.82 × 10^−3^	7.01 × 10^−2^	14.5 × 10^−1^
*C*4	1.13 × 10^−3^	0.74 × 10^−2^	1.38 × 10^−1^	1.55 × 10^−3^	1.36 × 10^−2^	2.79 × 10^−1^

**Table 8:** Performance indexes under parametric uncertainties of Set2.

	Boom Cylinder			Arm Cylinder		
	SAPE	IAE	ITAE	SAPE	IAE	ITAE
*C*1	8.91 × 10^−3^	10.8 × 10^−2^	21.3 × 10^−1^	3.75 × 10^−3^	13.1 × 10^−3^	2.65 × 10^−1^
*C*2	5.21 × 10^−3^	5.70 × 10^−2^	11.2 × 10^−1^	3.40 × 10^−3^	9.88 × 10^−3^	2.00 × 10^−1^
*C*3	4.58 × 10^−3^	7.10 × 10^−2^	13.9 × 10^−1^	3.06 × 10^−3^	9.17 × 10^−3^	1.87 × 10^−1^
*C*4	1.25 × 10^−3^	1.25 × 10^−2^	2.36 × 10^−1^	2.88 × 10^−3^	6.08 × 10^−3^	1.21 × 10^−1^

## Data Availability

Data are available on request due to restrictions e.g., privacy or ethical. The data presented in this study are available on request from the corresponding author. The data are not publicly available due to the presence of some confidential information.
